# An updated overview of *e-cigarette* impact on human health

**DOI:** 10.1186/s12931-021-01737-5

**Published:** 2021-05-18

**Authors:** Patrice Marques, Laura Piqueras, Maria-Jesus Sanz

**Affiliations:** 1 Department of Pharmacology, Faculty of Medicine, University of Valencia, Avda. Blasco Ibañez 15, 46010 Valencia, Spain; 2 Institute of Health Research INCLIVA, University Clinic Hospital of Valencia, Valencia, Spain; 3 CIBERDEM-Spanish Biomedical Research Centre in Diabetes and Associated Metabolic Disorders, ISCIII, Av. Monforte de Lemos 3-5, 28029 Madrid, Spain

**Keywords:** Electronic cigarette, E-cigarette, Nicotine, Tobacco, Humectants, Flavourings, Toxicity, Smoking cessation tool, COVID-19

## Abstract

The electronic cigarette (*e-cigarette*), for many considered as a safe alternative to conventional cigarettes, has revolutionised the tobacco industry in the last decades. In *e-cigarettes*, tobacco combustion is replaced by *e-liquid* heating, leading some manufacturers to propose that *e-cigarettes* have less harmful respiratory effects than tobacco consumption. Other innovative features such as the adjustment of nicotine content and the choice of pleasant flavours have won over many users. Nevertheless, the safety of *e-cigarette* consumption and its potential as a smoking cessation method remain controversial due to limited evidence. Moreover, it has been reported that the heating process itself can lead to the formation of new decomposition compounds of questionable toxicity. Numerous in vivo and in vitro studies have been performed to better understand the impact of these new inhalable compounds on human health. Results of toxicological analyses suggest that *e-cigarettes* can be safer than conventional cigarettes, although harmful effects from short-term *e-cigarette* use have been described. Worryingly, the potential long-term effects of *e-cigarette* consumption have been scarcely investigated. In this review, we take stock of the main findings in this field and their consequences for human health including coronavirus disease 2019 (COVID-19).

## Background

**Fig. 1:**
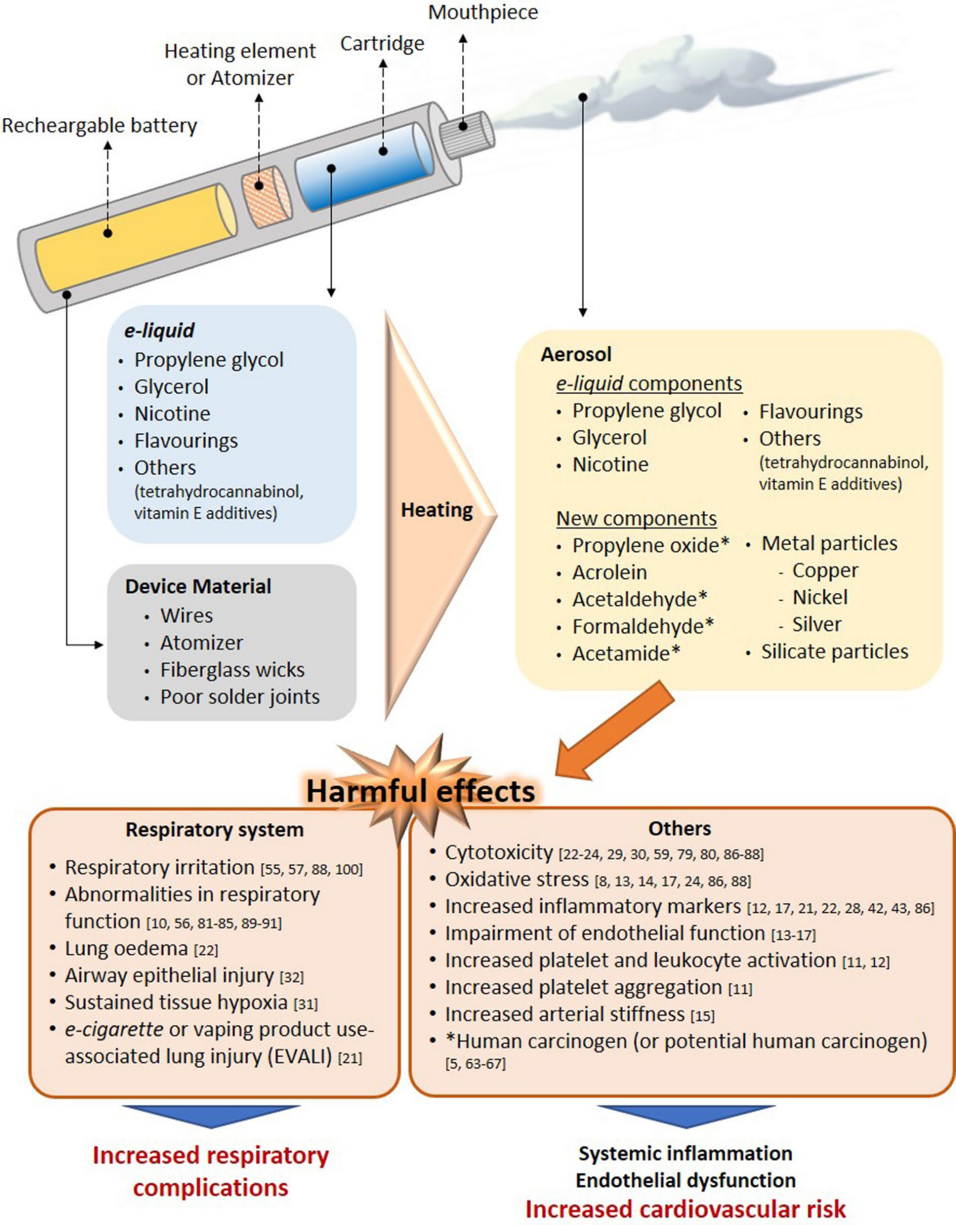
Effect of the heating process on aerosol composition. Main harmful effects documented. Several compounds detected in *e-cigarette* aerosols are not present in *e-liquid*s and the device material also seems to contribute to the presence of metal and silicate particles in the aerosols. The heating conditions especially on humectants, flavourings and the low-quality material used have been identified as the generator of the new compounds in aerosols. Some compounds generated from humectants (propylene glycol and glycerol) and flavourings, have been associated with clear airways impact, inflammation, impairment of cardiovascular function and toxicity. In addition, some of them are carcinogens or potential carcinogens

Electronic nicotine dispensing systems (ENDS), commonly known as electronic cigarettes or *e-cigarettes*, have been popularly considered a less harmful alternative to conventional cigarette smoking since they first appeared on the market more than a decade ago. *E-cigarettes* are electronic devices, essentially consisting of a cartridge, filled with an *e-liquid,* a heating element/atomiser necessary to heat the *e-liquid* to create a vapour that can be inhaled through a mouthpiece, and a rechargeable battery (Fig. 1) [1, 2]. Both the electronic devices and the different *e-liquids* are easily available in shops or online stores.

The *e-liquid* typically contains humectants and flavourings, with or without nicotine; once vapourised by the atomiser, the aerosol (vapour) provides a sensation similar to tobacco smoking, but purportedly without harmful effects [3]. However, it has been reported that the heating process can lead to the generation of new decomposition compounds that may be hazardous [4, 5]. The levels of nicotine, which is the key addictive component of tobacco, can also vary between the commercially available *e-liquids,* and even nicotine-free options are available. For this particular reason, *e-cigarettes* are often viewed as a smoking cessation tool, given that those with nicotine can prevent smoking craving, yet this idea has not been fully demonstrated [2, 6, 7].

Because *e-cigarettes* are combustion-free, and because most of the damaging and well-known effects of tobacco are derived from this reaction, there is a common and widely spread assumption that *e-cigarette* consumption or “vaping” is safer than conventional cigarette smoking. However, are they risk-free? Is there sufficient toxicological data on all the components employed in *e-liquids*? Do we really know the composition of the inhaled vapour during the heating process and its impact on health? Can *e-cigarettes* be used to curb tobacco use? Do their consumption impact on coronavirus disease 2019 (COVID-19)? In the present review, we have attempted to clarify these questions based on the existing scientific literature, and we have compiled new insights related with the toxicity derived from the use of these devices.

### Effect of *e-cigarette* vapour *versus* conventional cigarette exposure: in vivo and in vitro effects

Numerous studies have been performed to evaluate the safety/toxicity of *e-cigarette* use both in vivo and in in vitro cell culture.

One of the first studies in humans involved the analysis of 9 volunteers that consumed *e-cigarettes*, with or without nicotine, in a ventilated room for 2 h [8]. Pollutants in indoor air, exhaled nitric oxide (NO) and urinary metabolite profiles were analysed. The results of this acute experiment revealed that *e-cigarettes* are not emission-free, and ultrafine particles formed from propylene glycol (PG) could be detected in the lungs. The study also suggested that the presence of nicotine in *e-cigarettes* increased the levels of NO exhaled from consumers and provoked marked airway inflammation; however, no differences were found in the levels of exhaled carbon monoxide (CO), an oxidative stress marker, before and after *e-cigarette* consumption [8]. A more recent human study detected significantly higher levels of metabolites of hazardous compounds including benzene, ethylene oxide, acrylonitrile, acrolein and acrylamide in the urine of adolescent dual users (*e-cigarettes* and conventional tobacco consumers) than in adolescent *e-cigarette*-only users (Table 1) [9]. Moreover, the urine levels of metabolites of acrylonitrile, acrolein, propylene oxide, acrylamide and crotonaldehyde, all of which are detrimental for human health, were significantly higher in *e-cigarette*-only users than in non-smoker controls, reaching up to twice the registered values of those from non-smoker subjects (Table 1) [9]. In line with these observations, dysregulation of lung homeostasis has been documented in non-smokers subjected to acute inhalation of *e-cigarette* aerosols [10].

**Table 1:** Urine levels of metabolites of hazardous compounds in *e-cigarette*-only users versus dual users and non-smokers

Hazardous compounds	*E-Cigarette*–only users		Dual users		Non-smoker controls	
	Median	Range	Median	Range	Median	Range
PMA (ng/mg of creatinine; benzene)	0	0–2.0	0.2**	0–2.4	0	0–0.1
HEMA (ng/mg of creatinine; ethylene oxide)	0.5	0–7.6	1.0*	0–8.2	1.3	0–4.0
CNEMA (ng/mg of creatinine; acrylonitrile)	1.3	0–108.4	59.4**	3.7–142.6	0**	0–1.6
3-HPMA (ng/mg of creatinine; acrolein)	254.3	0–2311.6	439.7*	153.6–814.4	192.8*	0–1416.4
2-HPMA (ng/mg of creatinine; propylene oxide)	28.8	0–1382.6	40.2	10.2–310.9	15.2**	0–34.5
AAMA (ng/mg of creatinine; acrylamide)	67.3	0–581.2	235.6**	41.4–574.7	34.5**	0–182.0
HMPMA (ng/mg of creatinine; crotonaldehyde)	148.7	0–793.4	185.4	110.0–437.9	100.4*	0–522.1

The concentrations of metabolites were normalised to creatinine values. *PMA* phenylmercapturic acid (metabolite of benzene), *HEMA* 2-hydroxyethylmercapturic acid (metabolite of ethylene oxide), *CNEMA* 2 cyanoethylmercapturic acid (metabolite of acrylonitrile), *3-HPMA* 3 hydroxypropylmercapturic acid (metabolite of acrolein), *2-HPMA* 2-hydroxypropylmercapturic acid (metabolite of propylene oxide), *AAMA* 2-carbamoylethylmercapturic acid (metabolite of acrylamide), *HMPMA* 3-hydroxy-1-methylpropylmercapturic acid (metabolite of crotonaldehyde)

*P < 0.05 or **P < 0.01 versus *e-cigarette*–only users’ group. Data adapted from Rubinstein et al. [9]

Little is known about the effect of vaping on the immune system. Interestingly, both traditional and *e-cigarette* consumption by non-smokers was found to provoke short-term effects on platelet function, increasing platelet activation (levels of soluble CD40 ligand and the adhesion molecule P-selectin) and platelet aggregation, although to a lesser extent with *e-cigarettes* [11]. As found with platelets, the exposure of neutrophils to *e-cigarette* aerosol resulted in increased CD11b and CD66b expression being both markers of neutrophil activation [12]. Additionally, increased oxidative stress, vascular endothelial damage, impaired endothelial function, and changes in vascular tone have all been reported in different human studies on vaping [13–17]. In this context, it is widely accepted that platelet and leukocyte activation as well as endothelial dysfunction are associated with atherogenesis and cardiovascular morbidity [18, 19]. In line with these observations the potential association of daily *e-cigarettes* consumption and the increased risk of myocardial infarction remains controversial but benefits may occur when switching from tobacco to chronic *e-cigarette* use in blood pressure regulation, endothelial function and vascular stiffness (reviewed in [20]). Nevertheless, whether or not *e-cigarette* vaping has cardiovascular consequences requires further investigation.

More recently, in August 2019, the US Centers for Disease Control and Prevention (CDC) declared an outbreak of the *e-cigarette* or vaping product use-associated lung injury (EVALI) which caused several deaths in young population (reviewed in [20]). Indeed, computed tomography (CT scan) revealed local inflammation that impaired gas exchange caused by aerosolised oils from *e-cigarettes* [21]. However, most of the reported cases of lung injury were associated with use of *e-cigarettes* for tetrahydrocannabinol (THC) consumption as well as vitamin E additives [20] and not necessarily attributable to other *e-cigarette* components.

On the other hand, in a comparative study of mice subjected to either lab air, *e-cigarette* aerosol or cigarette smoke (CS) for 3 days (6 h-exposure per day), those exposed to *e-cigarette* aerosols showed significant increases in interleukin (IL)-6 but normal lung parenchyma with no evidence of apoptotic activity or elevations in IL-1β or tumour necrosis factor-α (TNFα) [22]. By contrast, animals exposed to CS showed lung inflammatory cell infiltration and elevations in inflammatory marker expression such as IL-6, IL-1β and TNFα [22]. Beyond airway disease, exposure to aerosols from *e-liquids* with or without nicotine has also been also associated with neurotoxicity in an early-life murine model [23].

Results from in vitro studies are in general agreement with the limited number of in vivo studies. For example, in an analysis using primary human umbilical vein endothelial cells (HUVEC) exposed to 11 commercially-available vapours, 5 were found to be acutely cytotoxic, and only 3 of those contained nicotine [24]. In addition, 5 of the 11 vapours tested (including 4 that were cytotoxic) reduced HUVEC proliferation and one of them increased the production of intracellular reactive oxygen species (ROS) [24]. Three of the most cytotoxic vapours—with effects similar to those of conventional high-nicotine CS extracts—also caused comparable morphological changes [24]. Endothelial cell migration is an important mechanism of vascular repair than can be disrupted in smokers due to endothelial dysfunction [25, 26]. In a comparative study of CS and *e-cigarette* aerosols, Taylor et al*.* found that exposure of HUVEC to *e-cigarette* aqueous extracts for 20 h did not affect migration in a scratch wound assay [27], whereas equivalent cells exposed to CS extract showed a significant inhibition in migration that was concentration dependent [27].

In cultured human airway epithelial cells, both *e-cigarette* aerosol and CS extract induced IL-8/CXCL8 (neutrophil chemoattractant) release [28]. In contrast, while CS extract reduced epithelial barrier integrity (determined by the translocation of dextran from the apical to the basolateral side of the cell layer), *e-cigarette* aerosol did not, suggesting that only CS extract negatively affected host defence [28]. Moreover, Higham et al*.* also found that *e-cigarette* aerosol caused IL-8/CXCL8 and matrix metallopeptidase 9 (MMP-9) release together with enhanced activity of elastase from neutrophils [12] which might facilitate neutrophil migration to the site of inflammation [12].

In a comparative study, repeated exposure of human gingival fibroblasts to CS condensate or to nicotine-rich or nicotine-free *e-vapour* condensates led to alterations in morphology, suppression of proliferation and induction of apoptosis, with changes in all three parameters greater in cells exposed to CS condensate [29]. Likewise, both *e-cigarette* aerosol and CS extract increased cell death in adenocarcinomic human alveolar basal epithelial cells (A549 cells), and again the effect was more damaging with CS extract than with *e-cigarette* aerosol (detrimental effects found at 2 mg/mL of CS extract vs. 64 mg/mL of *e-cigarette* extract) [22], which is in agreement with another study examining battery output voltage and cytotoxicity [30].

**Fig. 2:**
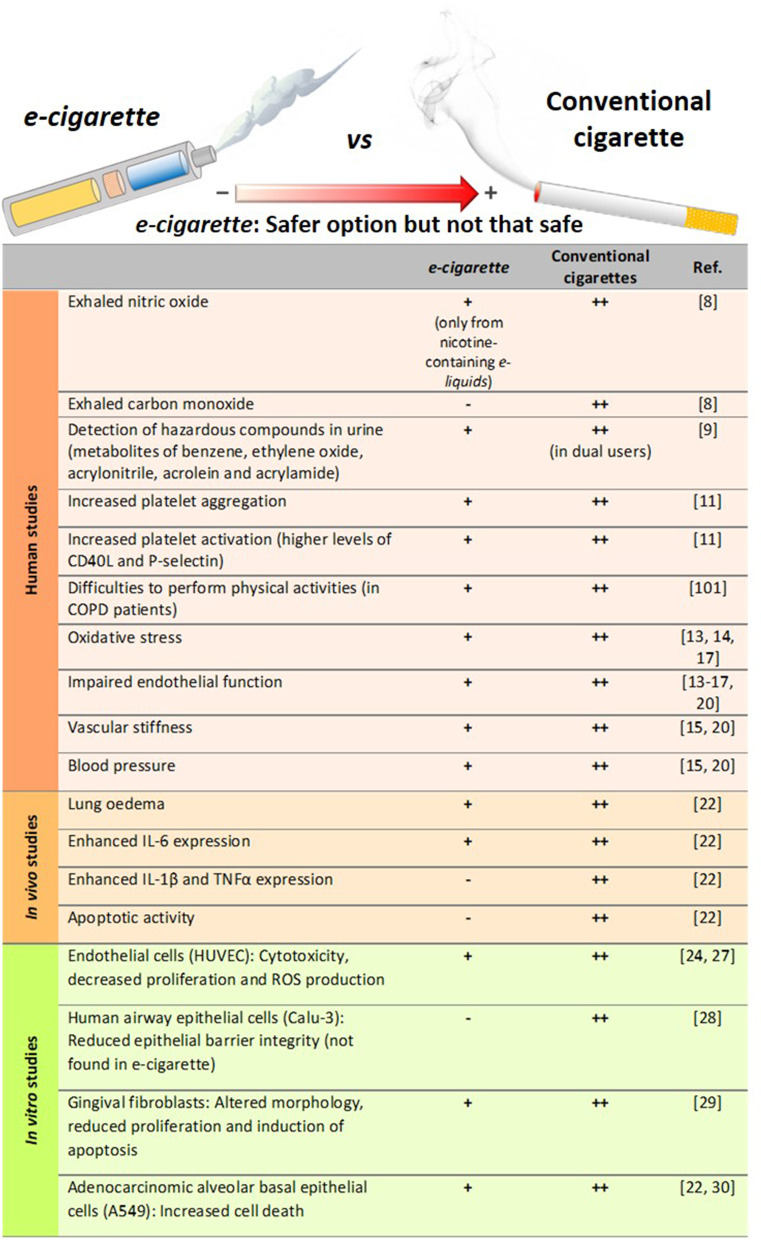
Comparison of the degree of harmful effects documented from *e-cigarette* and conventional cigarette consumption. Human studies, in vivo mice exposure and in vitro studies. All of these effects from *e-cigarettes* were documented to be lower than those exerted by conventional cigarettes, which may suggest that *e-cigarette* consumption could be a safer option than conventional tobacco smoking but not a clear safe choice

All this evidence would suggest that *e-cigarettes* are potentially less harmful than conventional cigarettes (Fig. 2) [11, 14, 22, 24, 27–29]. Importantly, however, most of these studies have investigated only short-term effects [10, 14, 15, 22, 27–29, 31, 32], and the long-term effects of *e-cigarette* consumption on human health are still unclear and require further study.

### Consequences of nicotine content

Beyond flavour, one of the major issues in the *e-liquid* market is the range of nicotine content available. Depending on the manufacturer, the concentration of this alkaloid can be presented as *low*, *medium* or *high*, or expressed as mg/mL or as a percentage (% v/v). The concentrations range from 0 (0%, nicotine-free option) to 20 mg/mL (2.0%)—the maximum nicotine threshold according to directive 2014/40/EU of the European Parliament and the European Union Council [33, 34]. Despite this normative, however, some commercial *e-liquids* have nicotine concentrations close to 54 mg/mL [35], much higher than the limits established by the European Union.

The mislabelling of nicotine content in *e-liquids* has been previously addressed [8, 34]. For instance, gas chromatography with a flame ionisation detector (GC-FID) revealed inconsistencies in the nicotine content with respect to the manufacturer´s declaration (average of 22 ± 0.8 mg/mL vs. 18 mg/mL) [8], which equates to a content ~ 22% higher than that indicated in the product label. Of note, several studies have detected nicotine in those *e-liquids* labelled as nicotine-free [5, 35, 36]. One study detected the presence of nicotine (0.11–6.90 mg/mL) in 5 of 23 nicotine-free labelled *e-liquids* by nuclear magnetic resonance spectroscopy [35], and another study found nicotine (average 8.9 mg/mL) in 13.6% (17/125) of the nicotine-free *e-liquids* as analysed by high performance liquid chromatography (HPLC) [36]. Among the 17 samples tested in this latter study 14 were identified to be counterfeit or suspected counterfeit. A third study detected nicotine in 7 of 10 nicotine-free refills, although the concentrations were lower than those identified in the previous analyses (0.1–15 µg/mL) [5]. Not only is there evidence of mislabelling of nicotine content among refills labelled as nicotine-free, but there also seems to be a history of poor labelling accuracy in nicotine-containing *e-liquids* [37, 38].

A comparison of the serum levels of nicotine from *e-cigarette* or conventional cigarette consumption has been recently reported [39]. Participants took one vape from an *e-cigarette*, with at least 12 mg/mL of nicotine, or inhaled a conventional cigarette, every 20 s for 10 min. Blood samples were collected 1, 2, 4, 6, 8, 10, 12 and 15 min after the first puff, and nicotine serum levels were measured by liquid chromatography-mass spectrometry (LC–MS). The results revealed higher serum levels of nicotine in the conventional CS group than in the *e-cigarette* group (25.9 ± 16.7 ng/mL vs. 11.5 ± 9.8 ng/mL). However, *e-cigarettes* containing 20 mg/mL of nicotine are more equivalent to normal cigarettes, based on the delivery of approximately 1 mg of nicotine every 5 min [40].

In this line, a study compared the acute impact of CS vs. *e-cigarette* vaping with equivalent nicotine content in healthy smokers and non-smokers. Both increased markers of oxidative stress and decreased NO bioavailability, flow-mediated dilation, and vitamin E levels showing no significant differences between tobacco and *e-cigarette* exposure (reviewed in [20]). Inasmuch, short-term *e-cigarette* use in healthy smokers resulted in marked impairment of endothelial function and an increase in arterial stiffness (reviewed in [20]). Similar effects on endothelial dysfunction and arterial stiffness were found in animals when they were exposed to *e-cigarette* vapor either for several days or chronically (reviewed in [20]). In contrast, other studies found acute microvascular endothelial dysfunction, increased oxidative stress and arterial stiffness in smokers after exposure to *e-cigarettes* with nicotine, but not after *e-cigarettes* without nicotine (reviewed in [20]). In women smokers, a study found a significant difference in stiffness after smoking just one tobacco cigarette, but not after use *of e-cigarettes* (reviewed in [20]).

It is well known that nicotine is extremely addictive and has a multitude of harmful effects. Nicotine has significant biologic activity and adversely affects several physiological systems including the cardiovascular, respiratory, immunological and reproductive systems, and can also compromise lung and kidney function [41]. Recently, a sub-chronic whole-body exposure of *e-liquid* (2 h/day, 5 days/week, 30 days) containing PG alone or PG with nicotine (25 mg/mL) to wild type (WT) animals or knockout (KO) mice in α7 nicotinic acetylcholine receptor (nAChRα7-KO) revealed a partly nAChRα7-dependent lung inflammation [42]. While sub-chronic exposure to PG/nicotine promote nAChRα7-dependent increased levels of different cytokines and chemokines in the bronchoalveolar lavage fluid (BALF) such as IL-1α, IL-2, IL-9, interferon γ (IFNγ), granulocyte-macrophage colony-stimulating factor (GM-CSF), monocyte chemoattractant protein-1 (MCP-1/CCL2) and regulated on activation, normal T cell expressed and secreted (RANTES/CCL5), the enhanced levels of IL-1β, IL-5 and TNFα were nAChRα7 independent. In general, most of the cytokines detected in BALF were significantly increased in WT mice exposed to PG with nicotine compared to PG alone or air control [42]. Some of these effects were found to be through nicotine activation of NF-κB signalling albeit in females but not in males. In addition, PG with nicotine caused increased macrophage and CD4^+^/CD8^+^ T-lymphocytes cell counts in BALF compared to air control, but these effects were ameliorated when animals were sub-chronically exposed to PG alone [42].

Of note, another study indicated that although RANTES/CCL5 and CCR1 mRNA were upregulated in flavour/nicotine-containing *e-cigarette* users, vaping flavour and nicotine-less *e-cigarettes* did not significantly dysregulate cytokine and inflammasome activation [43].

In addition to its toxicological effects on foetus development, nicotine can disrupt brain development in adolescents and young adults [44–46]. Several studies have also suggested that nicotine is potentially carcinogenic (reviewed in [41]), but more work is needed to prove its carcinogenicity independently of the combustion products of tobacco [47]. In this latter regard, no differences were encountered in the frequency of tumour appearance in rats subjected to long-term (2 years) inhalation of nicotine when compared with control rats [48]. Despite the lack of carcinogenicity evidence, it has been reported that nicotine promotes tumour cell survival by decreasing apoptosis and increasing proliferation [49], indicating that it may work as a “tumour enhancer”. In a very recent study, chronic administration of nicotine to mice (1 mg/kg every 3 days for a 60-day period) enhanced brain metastasis by skewing the polarity of M2 microglia, which increases metastatic tumour growth [50]. Assuming that a conventional cigarette contains 0.172–1.702 mg of nicotine [51], the daily nicotine dose administered to these animals corresponds to 40–400 cigarettes for a 70 kg-adult, which is a dose of an extremely heavy smoker. We would argue that further studies with chronic administration of low doses of nicotine are required to clearly evaluate its impact on carcinogenicity.

In the aforementioned study exposing human gingival fibroblasts to CS condensate or to nicotine-rich or nicotine-free *e-vapour* condensates [29], the detrimental effects were greater in cells exposed to nicotine-rich condensate than to nicotine-free condensate, suggesting that the possible injurious effects of nicotine should be considered when purchasing *e-refills*. It is also noteworthy that among the 3 most cytotoxic vapours for HUVEC evaluated in the Putzhammer et al*.* study, 2 were nicotine-free, which suggests that nicotine is not the only hazardous component in *e-cigarettes* [24]*.*

The lethal dose of nicotine for an adult is estimated at 30–60 mg [52]. Given that nicotine easily diffuses from the dermis to the bloodstream, acute nicotine exposure by *e-liquid* spilling (5 mL of a 20 mg/mL nicotine-containing refill is equivalent to 100 mg of nicotine) can easily be toxic or even deadly [8]. Thus, devices with rechargeable refills are another issue of concern with *e-cigarettes*, especially when *e-liquids* are not sold in child-safe containers, increasing the risk of spilling, swallowing or breathing.

These data overall indicate that the harmful effects of nicotine should not be underestimated. Despite the established regulations, some inaccuracies in nicotine content labelling remain in different brands of *e-liquids*. Consequently, stricter regulation and a higher quality control in the *e-liquid* industry are required.

### Effect of humectants and their heating-related products

In this particular aspect, again the composition of the *e-liquid* varies significantly among different commercial brands [4, 35]. The most common and major components of *e-liquids* are PG or 1,2-propanediol, and glycerol or glycerine (propane-1,2,3-triol). Both types of compounds are used as humectants to prevent the *e-liquid* from drying out [2, 53] and are classified by the Food and Drug Administration (FDA) as *“Generally Recognised as Safe”* [54]. In fact, they are widely used as alimentary and pharmaceutical products [2]. In an analysis of 54 commercially available *e-liquids*, PG and glycerol were detected in almost all samples at concentrations ranging from 0.4% to 98% (average 57%) and from 0.3% to 95% (average 37%), respectively [35].

With regards to toxicity, little is known about the effects of humectants when they are heated and chronically inhaled. Studies have indicated that PG can induce respiratory irritation and increase the probability of asthma development [55, 56], and both PG and glycerol from *e-cigarettes* might reach concentrations sufficiently high to potentially cause irritation of the airways [57]. Indeed, the latter study established that one *e-cigarette* puff results in a PG exposure of 430–603 mg/m^3^, which is higher than the levels reported to cause airway irritation (average 309 mg/m^3^) based on a human study [55]. The same study established that one *e-cigarette* puff results in a glycerol exposure of 348–495 mg/m^3^ [57], which is close to the levels reported to cause airway irritation in rats (662 mg/m^3^) [58].

Airway epithelial injury induced by acute vaping of PG and glycerol aerosols (50:50 vol/vol), with or without nicotine, has been reported in two randomised clinical trials in young tobacco smokers [32]. In vitro, aerosols from glycerol only-containing refills showed cytotoxicity in A549 and human embryonic stem cells, even at a low battery output voltage [59]. PG was also found to affect early neurodevelopment in a zebrafish model [60]. Another important issue is that, under heating conditions PG can produce acetaldehyde or formaldehyde (119.2 or 143.7 ng/puff at 20 W, respectively, on average), while glycerol can also generate acrolein (53.0, 1000.0 or 5.9 ng/puff at 20 W, respectively, on average), all carbonyls with a well-documented toxicity [61]. Although, assuming 15 puffs per *e-cigarette* unit, carbonyls produced by PG or glycerol heating would be below the maximum levels found in a conventional cigarette combustion (Table 2) [51, 62]. Nevertheless, further studies are required to properly test the deleterious effects of all these compounds at physiological doses resembling those to which individuals are chronically exposed.

**Table 2:** Content comparison of the most common carbonyl compounds from *e-cigarettes* versus conventional tobacco cigarettes consumption

	Formaldehyde (μg)	Acetaldehyde (μg)	Acrolein (μg)	References
*E-cigarette* (unit = 15 puffs)	0.2–5.61	0.11–1.36	0.07–9	[4, 68]
Conventional cigarette (unit)	1.6–52.1	52–828	2.4–98.2	[51, 62]

Although PG and glycerol are the major components of *e-liquids* other components have been detected. When the aerosols of 4 commercially available *e-liquids* chosen from a top 10 list of “*Best E-Cigarettes of 2014”*, were analysed by gas chromatography-mass spectrometry (GC–MS) after heating, numerous compounds were detected, with nearly half of them not previously identified [4], thus suggesting that the heating process per se generates new compounds of unknown consequence. Of note, the analysis identified formaldehyde, acetaldehyde and acrolein [4], 3 carbonyl compounds with known high toxicity [63–67]. While no information was given regarding formaldehyde and acetaldehyde concentrations, the authors calculated that one puff could result in an acrolein exposure of 0.003–0.015 μg/mL [4]. Assuming 40 mL per puff and 15 puffs per *e-cigarette* unit (according to several manufacturers) [4], each *e-cigarette* unit would generate approximately 1.8–9 μg of acrolein, which is less than the levels of acrolein emitted by a conventional tobacco cigarette (18.3–98.2 μg) [51]. However, given that *e-cigarette* units of vaping are not well established, users may puff intermittently throughout the whole day. Thus, assuming 400 to 500 puffs per cartridge, users could be exposed to up to 300 μg of acrolein.

In a similar study, acrolein was found in 11 of 12 aerosols tested, with a similar content range (approximately 0.07–4.19 μg per *e-cigarette* unit) [68]. In the same study, both formaldehyde and acetaldehyde were detected in all of the aerosols tested, with contents of 0.2–5.61 μg and 0.11–1.36 μg, respectively, per *e-cigarette* unit [68]. It is important to point out that the levels of these toxic products in *e-cigarette* aerosols are significantly lower than those found in CS: 9 times lower for formaldehyde, 450 times lower for acetaldehyde and 15 times lower for acrolein (Table 2) [62, 68].

Other compounds that have been detected in aerosols include acetamide, a potential human carcinogen [5], and some aldehydes [69], although their levels were minimal. Interestingly, the existence of harmful concentrations of diethylene glycol, a known cytotoxic agent, in *e-liquid* aerosols is contentious with some studies detecting its presence [4, 68, 70–72], and others finding low subtoxic concentrations [73, 74]. Similar observations were reported for the content ethylene glycol. In this regard, either it was detected at concentrations that did not exceed the authorised limit [73], or it was absent from the aerosols produced [4, 71, 72]. Only one study revealed its presence at high concentration in a very low number of samples [5]. Nevertheless, its presence above 1 mg/g is not allowed by the FDA [73]. Figure 1 lists the main compounds detected in aerosols derived from humectant heating and their potential damaging effects. It would seem that future studies should analyse the possible toxic effects of humectants and related products at concentrations similar to those that *e-cigarette* vapers are exposed to reach conclusive results.

### Impact of flavouring compounds

The range of *e-liquid* flavours available to consumers is extensive and is used to attract both current smokers and new *e-cigarette* users, which is a growing public health concern [6]. In fact, over 5 million middle- and high-school students were current users of *e-cigarettes* in 2019 [75], and appealing flavours have been identified as the primary reason for *e-cigarette* consumption in 81% of young users [76]. Since 2016, the FDA regulates the flavours used in the *e-cigarette* market and has recently published an enforcement policy on unauthorised flavours, including fruit and mint flavours, which are more appealing to young users [77]. However, the long-term effects of all flavour chemicals used by this industry (which are more than 15,000) remain unknown and they are not usually included in the product label [78]. Furthermore, there is no safety guarantee since they may harbour potential toxic or irritating properties [5].

With regards to the multitude of available flavours, some have demonstrated cytotoxicity [59, 79]. Bahl et al. evaluated the toxicity of 36 different *e-liquids* and 29 different flavours on human embryonic stem cells, mouse neural stem cells and human pulmonary fibroblasts using a metabolic activity assay. In general, those *e-liquids* that were bubblegum-, butterscotch- and caramel-flavoured did not show any overt cytotoxicity even at the highest dose tested. By contrast, those *e-liquids* with *Freedom Smoke Menthol Arctic* and *Global Smoke Caramel* flavours had marked cytotoxic effects on pulmonary fibroblasts and those with *Cinnamon Ceylon* flavour were the most cytotoxic in all cell lines [79]. A further study from the same group [80] revealed that high cytotoxicity is a recurrent feature of cinnamon-flavoured *e-liquids.* In this line, results from GC–MS and HPLC analyses indicated that cinnamaldehyde (CAD) and 2-methoxycinnamaldehyde, but not dipropylene glycol or vanillin, were mainly responsible for the high cytotoxicity of cinnamon-flavoured *e-liquids* [80]. Other flavouring-related compounds that are associated with respiratory complications [81–83], such as diacetyl, 2,3-pentanedione or acetoin, were found in 47 out of 51 aerosols of flavoured *e-liquids* tested [84]*.* Allen et al*.* calculated an average of 239 μg of diacetyl per cartridge [84]. Assuming again 400 puffs per cartridge and 40 mL per puff, is it is possible to estimate an average of 0.015 ppm of diacetyl per puff, which could compromise normal lung function in the long-term [85].

The cytotoxic and pro-inflammatory effects of different *e-cigarette* flavouring chemicals were also tested on two human monocytic cell lines—mono mac 6 (MM6) and U937 [86]. Among the flavouring chemicals tested, CAD was found to be the most toxic and O-vanillin and pentanedione also showed significant cytotoxicity; by contrast, acetoin, diacetyl, maltol, and coumarin did not show any toxicity at the concentrations assayed (10–1000 µM). Of interest, a higher toxicity was evident when combinations of different flavours or mixed equal proportions of *e-liquids* from 10 differently flavoured *e-liquids* were tested, suggesting that vaping a single flavour is less toxic than inhaling mixed flavours [86]. Also, all the tested flavours produced significant levels of ROS in a cell-free ROS production assay. Finally, diacetyl, pentanedione, O-vanillin, maltol, coumarin, and CAD induced significant IL-8 secretion from MM6 and U937 monocytes [86]. It should be borne in mind, however, that the concentrations assayed were in the supra-physiological range and it is likely that, once inhaled, these concentrations are not reached in the airway space. Indeed, one of the limitations of the study was that human cells are not exposed to *e-liquids *per se, but rather to the aerosols where the concentrations are lower [86]. In this line, the maximum concentration tested (1000 µM) would correspond to approximately 80 to 150 ppm, which is far higher than the levels found in aerosols of some of these compounds [84]. Moreover, on a day-to-day basis, lungs of *e-cigarette* users are not constantly exposed to these chemicals for 24 h at these concentrations. Similar limitations were found when five of seven flavourings were found to cause cytotoxicity in human bronchial epithelial cells [87].

Recently, a commonly commercialized *crème brûlée*-flavoured aerosol was found to contain high concentrations of benzoic acid (86.9 μg/puff), a well-established respiratory irritant [88]. When human lung epithelial cells (BEAS-2B and H292) were exposed to this aerosol for 1 h, a marked cytotoxicity was observed in BEAS-2B but not in H292 cells, 24 h later. However, increased ROS production was registered in H292 cells [88].

Therefore, to fully understand the effects of these compounds, it is relevant the cell cultures selected for performing these assays, as well as the use of in vivo models that mimic the real-life situation of chronic *e-cigarette* vapers to clarify their impact on human health.

### The *e-cigarette* device

While the bulk of studies related to the impact of *e-cigarette* use on human health has focused on the *e-liquid* components and the resulting aerosols produced after heating, a few studies have addressed the material of the electronic device and its potential consequences—specifically, the potential presence of metals such as copper, nickel or silver particles in *e-liquids* and aerosols originating from the filaments and wires and the atomiser [89–91].

Other important components in the aerosols include silicate particles from the fiberglass wicks or silicone [89–91]. Many of these products are known to cause abnormalities in respiratory function and respiratory diseases [89–91], but more in-depth studies are required. Interestingly, the battery output voltage also seems to have an impact on the cytotoxicity of the aerosol vapours, with *e-liquids* from a higher battery output voltage showing more toxicity to A549 cells [30].

A recent study compared the acute effects of *e-cigarette* vapor (with PG/vegetable glycerine plus tobacco flavouring but without nicotine) generated from stainless‐steel atomizer (SS) heating element or from a nickel‐chromium alloy (NC) [92]. Some rats received a single *e-cigarette* exposure for 2 h from a NC heating element (60 or 70 W); other rats received a similar exposure of *e-cigarette* vapor using a SS heating element for the same period of time (60 or 70 W) and, a final group of animals were exposed for 2 h to air. Neither the air‐exposed rats nor those exposed to *e-cigarette* vapor using SS heating elements developed respiratory distress. In contrast, 80% of the rats exposed to *e-cigarette* vapor using NC heating units developed clinical acute respiratory distress when a 70‐W power setting was employed. Thus, suggesting that operating units at higher than recommended settings can cause adverse effects. Nevertheless, there is no doubt that the deleterious effects of battery output voltage are not comparable to those exerted by CS extracts [30] (Figs. 1 and 2).

### *E-cigarettes* as a smoking cessation tool

CS contains a large number of substances—about 7000 different constituents in total, with sizes ranging from atoms to particulate matter, and with many hundreds likely responsible for the harmful effects of this habit [93]. Given that tobacco is being substituted in great part by *e-cigarettes* with different chemical compositions, manufacturers claim that e*-cigarette* will not cause lung diseases such as lung cancer, chronic obstructive pulmonary disease, or cardiovascular disorders often associated with conventional cigarette consumption [3, 94]. However, the World Health Organisation suggests that *e-cigarettes* cannot be considered as a viable method to quit smoking, due to a lack of evidence [7, 95]. Indeed, the results of studies addressing the use of *e-cigarettes* as a smoking cessation tool remain controversial [96–100]. Moreover, both FDA and CDC are actively investigating the incidence of severe respiratory symptoms associated with the use of vaping products [77]. Because many *e-liquids* contain nicotine, which is well known for its powerful addictive properties [41], *e-cigarette* users can easily switch to conventional cigarette smoking, avoiding smoking cessation. Nevertheless, the possibility of vaping nicotine-free *e-cigarettes* has led to the branding of these devices as smoking cessation tools [2, 6, 7].

In a recently published randomised trial of 886 subjects who were willing to quit smoking [100], the abstinence rate was found to be twice as high in the *e-cigarette* group than in the nicotine-replacement group (18.0% vs. 9.9%) after 1 year. Of note, the abstinence rate found in the nicotine-replacement group was lower than what is usually expected with this therapy. Nevertheless, the incidence of throat and mouth irritation was higher in the *e-cigarette* group than in the nicotine-replacement group (65.3% vs. 51.2%, respectively). Also, the participant adherence to the treatment after 1-year abstinence was significantly higher in the *e-cigarette* group (80%) than in nicotine-replacement products group (9%) [100].

On the other hand, it is estimated that COPD could become the third leading cause of death in 2030 [101]. Given that COPD is generally associated with smoking habits (approximately 15 to 20% of smokers develop COPD) [101], smoking cessation is imperative among COPD smokers. Published data revealed a clear reduction of conventional cigarette consumption in COPD smokers that switched to *e-cigarettes* [101]. Indeed, a significant reduction in exacerbations was observed and, consequently, the ability to perform physical activities was improved when data was compared with those non-vapers COPD smokers. Nevertheless, a longer follow-up of these COPD patients is required to find out whether they have quitted conventional smoking or even vaping, since the final goal under these circumstances is to quit both habits.

Based on the current literature, it seems that several factors have led to the success of *e-cigarette* use as a smoking cessation tool. First, some *e-cigarette* flavours positively affect smoking cessation outcomes among smokers [102]. Second, *e-cigarettes* have been described to improve smoking cessation rate only among highly-dependent smokers and not among conventional smokers, suggesting that the individual degree of nicotine dependence plays an important role in this process [97]. Third, the general belief of their relative harmfulness to consumers' health compared with conventional combustible tobacco [103]. And finally, the exposure to point-of-sale marketing of *e-cigarette* has also been identified to affect the smoking cessation success [96].

### Implication of *e-cigarette* consumption in COVID-19 time

Different reports have pointed out that smokers and vapers are more vulnerable to SARS-CoV-2 (Severe Acute Respiratory Syndrome Coronavirus 2) infections or more prone to adverse outcomes if they suffer COVID-19 [104]. However, while a systematic review indicated that cigarette smoking is probably associated with enhanced damage from COVID-19, a meta-analysis did not, yet the latter had several limitations due to the small sample sizes [105].

Interestingly, most of these reports linking COVID-19 harmful effects with smoking or vaping, are based on their capability of increasing the expression of angiotensin-converting enzyme 2 (ACE2) in the lung. It is well known that ACE2 is the gate for SARS-CoV-2 entrance to the airways [106] and it is mainly expressed in type 2 alveolar epithelial cells and alveolar macrophages [107]. To date, most of the studies in this field indicate that current smokers have higher expression of ACE2 in the airways (reviewed by [108]) than healthy non-smokers [109, 110]. However, while a recent report indicated that *e-cigarette* vaping also caused nicotine-dependent ACE2 up-regulation [42], others have revealed that neither acute inhalation of *e-cigarette* vapour nor *e-cigarette* users had increased lung ACE2 expression regardless nicotine presence in the *e-liquid* [43, 110].

In regard to these contentions, current knowledge suggests that increased ACE2 expression is not necessarily linked to enhanced susceptibility to SARS-CoV-2 infection and adverse outcome. Indeed, elderly population express lower levels of ACE2 than young people and SARS-CoV-2/ACE2 interaction further decreases ACE2 expression. In fact, most of the deaths provoked by COVID-19 took place in people over 60 years old of age [111]. Therefore, it is plausible that the increased susceptibility to disease progression and the subsequent fatal outcome in this population is related to poor angiotensin 1-7 (Ang-1-7) generation, the main peptide generated by ACE2, and probably to their inaccessibility to its anti-inflammatory effects. Furthermore, it seems that all the efforts towards increasing ACE2 expression may result in a better resolution of the pneumonic process associated to this pandemic disease.

Nevertheless, additional complications associated to COVID-19 are increased thrombotic events and cytokine storm. In the lungs, *e-cigarette* consumption has been correlated to toxicity, oxidative stress, and inflammatory response [32, 112]. More recently, a study revealed that while the use of nicotine/flavour-containing *e-cigarettes* led to significant cytokine dysregulation and potential inflammasome activation, none of these effects were detected in non-flavoured and non-nicotine-containing *e-cigarettes* [43]. Therefore, taken together these observations, *e-cigarette* use may still be a potent risk factor for severe COVID-19 development depending on the flavour and nicotine content.

In summary, it seems that either smoking or nicotine vaping may adversely impact on COVID-19 outcome. However, additional follow up studies are required in COVID-19 pandemic to clarify the effect of *e-cigarette* use on lung and cardiovascular complications derived from SARS-CoV-2 infection.

## Conclusions

The harmful effects of CS and their deleterious consequences are both well recognised and widely investigated. However, and based on the studies carried out so far, it seems that *e-cigarette* consumption is less toxic than tobacco smoking. This does not necessarily mean, however, that *e-cigarettes* are free from hazardous effects. Indeed, studies investigating their long-term effects on human health are urgently required. In this regard, the main additional studies needed in this field are summarized in Table 3.

**Table 3:** Future research needed in the impact of *e-cigarette*-consumption in human health

Future research items to be addressed
Evaluate long-term effects of *e-cigarette*-consumption in human health for safety guarantee
Search for clear evidences of *e-cigarette* as a smoking cessation tool
Increase the number of in vivo and ex vivo studies (preferentially in humans)
Study the effects of *e-cigarette*-consumption on the immune system
Study effects the impact of *e-cigarette*-consumption on the cardiovascular system
Analyse potential toxicological effects of humectants, flavourings and related products after the heating process at physiological concentrations (similar to those that *e-cigarette* vapers are exposed)
Limit the number of flavourings authorised: The list should be strictly limited to those flavourings with long-term safety guaranteed, and appealing flavours for children/adolescents should be banned
Eradicate counterfeit products and implement a stricter regulation (e.g., Establish a strict range of nicotine content worldwide; standardize labelling; etc.)
Material device: all materials used should not generate harmful particles in aerosols
Follow-up study of the effects on respiratory and cardiovascular complications derived from SARS-CoV-2 infection

The composition of *e-liquids* requires stricter regulation, as they can be easily bought online and many incidences of mislabelling have been detected, which can seriously affect consumers’ health. Beyond their unknown long-term effects on human health, the extended list of appealing flavours available seems to attract new “never-smokers”, which is especially worrying among young users. Additionally, there is still a lack of evidence of *e-cigarette* consumption as a smoking cessation method. Indeed, *e-cigarettes* containing nicotine may relieve the craving for smoking, but not the conventional cigarette smoking habit.

Interestingly, there is a strong difference of opinion on *e-cigarettes* between countries. Whereas countries such as Brazil, Uruguay and India have banned the sale of *e-cigarettes*, others such as the United Kingdom support this device to quit smoking. The increasing number of adolescent users and reported deaths in the United States prompted the government to ban the sale of flavoured *e-cigarettes* in 2020. The difference in opinion worldwide may be due to different restrictions imposed. For example, while no more than 20 ng/mL of nicotine is allowed in the EU, *e-liquids* with 59 mg/dL are currently available in the United States. Nevertheless, despite the national restrictions, users can easily access foreign or even counterfeit products online.

In regard to COVID-19 pandemic, the actual literature suggests that nicotine vaping may display adverse outcomes. Therefore, follow up studies are necessary to clarify the impact of *e-cigarette* consumption on human health in SARS-CoV-2 infection.

In conclusion, *e-cigarettes* could be a good alternative to conventional tobacco cigarettes, with less side effects; however, a stricter sale control, a proper regulation of the industry including flavour restriction, as well as further toxicological studies, including their chronic effects, are warranted.

## Data Availability

Not applicable.

## Abbreviations

ACE2: Angiotensin-converting enzyme 2
Ang-1-7: Angiotensin 1-7
BALF: Bronchoalveolar lavage fluid
CAD: Cinnamaldehyde
CDC: US Centers for Disease Control and Prevention
CO: Carbon monoxide
COPD: Chronic obstructive pulmonary disease
COVID-19: Coronavirus disease 2019
CS: Cigarette smoke
ENDS: Electronic nicotine dispensing systems
EVALI: *e-cigarette* or vaping product use-associated lung injury
FDA: Food and Drug Administration
GC-FID: Gas chromatography with a flame ionisation detector
GC–MS: Gas chromatography-mass spectrometry
GM-CSF: Granulocyte–macrophage colony-stimulating factor
HPLC: High performance liquid chromatography
HUVEC: Human umbilical vein endothelial cells
IL: Interleukin
IFNγ: Interferon γ
KO: Knockout
LC–MS: Liquid chromatography-mass spectrometry
MCP-1/CCL2: Monocyte chemoattractant protein-1
MMP-9: Matrix metallopeptidase 9
nAChRα7: α7 Nicotinic acetylcholine receptor
NC: Nickel‐chromium alloy
NO: Nitric oxide
PG: Propylene glycol
RANTES/CCL5: Regulated on activation, normal T cell expressed and secreted
ROS: Reactive oxygen species
SARS-CoV-2: Severe acute respiratory syndrome coronavirus 2
SS: Stainless‐steel atomizer
THC: Tetrahydrocannabinol
TNFα: Tumour necrosis factor-α
WT: Wild type
